# Ultrasound-Guided Manual Therapy for Limitation of Knee Flexion Due to Quadriceps Contusion With Heterotopic Ossification: A Case Report

**DOI:** 10.7759/cureus.92299

**Published:** 2025-09-14

**Authors:** Akira Ogawa, Masashi Kawabata, Yuto Uchida, Yusuke Kumazawa

**Affiliations:** 1 Department of Rehabilitation, Kumazawa Orthopedics Clinic, Chofu, JPN; 2 Department of Rehabilitation, Kitasato University School of Allied Health Sciences, Sagamihara, JPN; 3 Department of Sports Medicine, Kitasato University Graduate School of Medical Sciences, Sagamihara, JPN; 4 Department of Orthopedics, Kumazawa Orthopedics Clinic, Chofu, JPN

**Keywords:** heterotopic ossification, limitation of knee flexion, myositis ossificans, rehabilitation, ultrasound-guided manual therapy

## Abstract

Heterotopic ossification (HO) is a known complication of quadriceps contusions that can lead to pain and limitation of knee flexion. Although conservative treatment is generally recommended, the therapeutic role of manual therapy in HO remains unclear.

We describe a 20-year-old collegiate soccer player who developed HO in the vastus intermedius after a quadriceps contusion, presenting with knee flexion limited to 120° (both active and passive) and severe pain (Numeric Rating Scale: NRS, 8/10). Dynamic ultrasonography revealed impaired tissue gliding. Ultrasound-guided manual therapy, including lifting and transverse gliding maneuvers, was performed. After the first session, knee flexion improved to 130° with pain relief (NRS, 3/10), and dynamic ultrasonography confirmed improved tissue gliding. With weekly rehabilitation and a structured home program, the patient achieved full pain-free flexion and running within one month, and returned to team training at two months.

Previous reports suggest that return to sport after conservative management of quadriceps HO may take up to 24 weeks. In contrast, this case demonstrated recovery within eight weeks, likely due to restoration of soft tissue mobility rather than reduction of the ossified lesion.

This case highlights the clinical novelty of ultrasound-guided manual therapy for HO, which has rarely been reported in the literature. Dynamic ultrasonography enables precise visualization of adhesions, directly enhancing the accuracy and effectiveness of manual therapy. This approach may be considered before surgical excision, particularly in athletes who require an early return to sport.

## Introduction

Heterotopic ossification (HO), also known as myositis ossificans (MO), often develops after muscle contusions sustained during sports activities [1]. Most cases in the lower extremities occur in the quadriceps femoris, particularly in the vastus intermedius. HO in this region can cause limitation of knee flexion and pain [1,2]. HO is a general term for ectopic bone formation in nonskeletal tissues and can be classified into hereditary and acquired types, involving diverse signaling pathways such as bone morphogenetic protein/transforming growth factor beta, Hedgehog, and Wnt, as well as multiple progenitor cells [3]. In contrast, MO is regarded as a localized subtype of acquired HO that develops after muscle contusion or strain and progresses through an endochondral ossification process [3,4]. Because MO is accompanied by muscle injury, fibrosis and scar formation are likely to occur during the repair process, which may lead not only to ectopic bone formation but also to impaired gliding due to adhesions with adjacent tissues [4,5].

Conservative management, including medication, physical therapy, and rehabilitation, is typically performed. Symptoms typically improve within six to eight weeks, with 90% of patients resuming light exercise by three months, 90% returning to sports by six months, and most regaining their preinjury competitive level within one year [6,7]. Surgical excision is considered in cases that are refractory to conservative treatment. To minimize recurrence, excision is recommended at least six months after the injury, once the lesion has matured [8]. Excision can alleviate symptoms and allow a return to sports within four to six weeks postoperatively [9].

Despite these established management strategies, few reports have addressed the specific rehabilitation interventions for HO, and the role of manual therapy in improving tissue mobility and function remains unclear. To our knowledge, similar applications of ultrasound-guided manual therapy for HO have rarely been reported. This case illustrates its potential to directly address adhesions, leading to pain reduction, restoration of range of motion (ROM), and earlier return to sports compared with previously reported conservative cases.

## Case presentation

**Figure 1:**
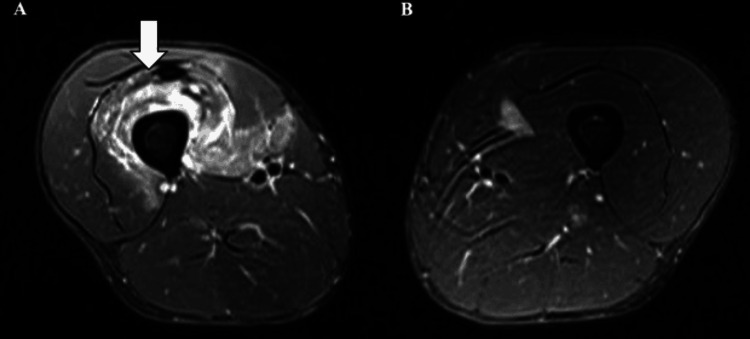
Axial short tau inversion recovery magnetic resonance imaging of the thigh at the initial presentation (A) Affected side. Arrow: A high signal intensity was observed within the vastus intermedius. (B) Unaffected side for comparison

**Figure 2:**
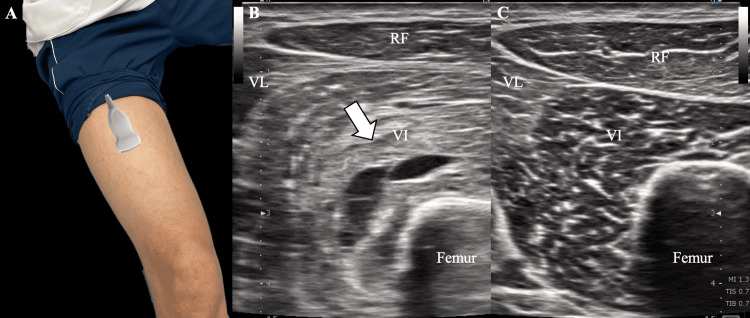
Ultrasound image of the thigh at initial presentation (A) The ultrasound probe was placed on the thigh as shown, and the obtained images are presented as ultrasound image of the thigh on the (B) affected side and (C) unaffected side Arrow: Hyperechoic and hypoechoic changes observed in the vastus intermedius. The hyperechoic changes were suggestive of tissue hardening, such as fibrosis associated with muscle injury, while the hypoechoic areas indicated possible hematoma or fluid collection RF: rectus femoris; VL: vastus lateralis; VI: vastus intermedius Image credit: The image was created and modified by the author Akira Ogawa (demonstration with a model)

A 20-year-old male collegiate soccer player sustained a direct blow to the anterior right thigh when an opponent's knee struck him during a match. He developed severe pain and swelling and visited a nearby clinic, where conservative management with rest and oral medication was initiated. However, pain and limitation of knee flexion persisted three months after injury, leading him to visit our hospital. Magnetic resonance imaging and ultrasound examination revealed a contusion of the vastus intermedius (Figures 1, 2).

**Figure 3:**
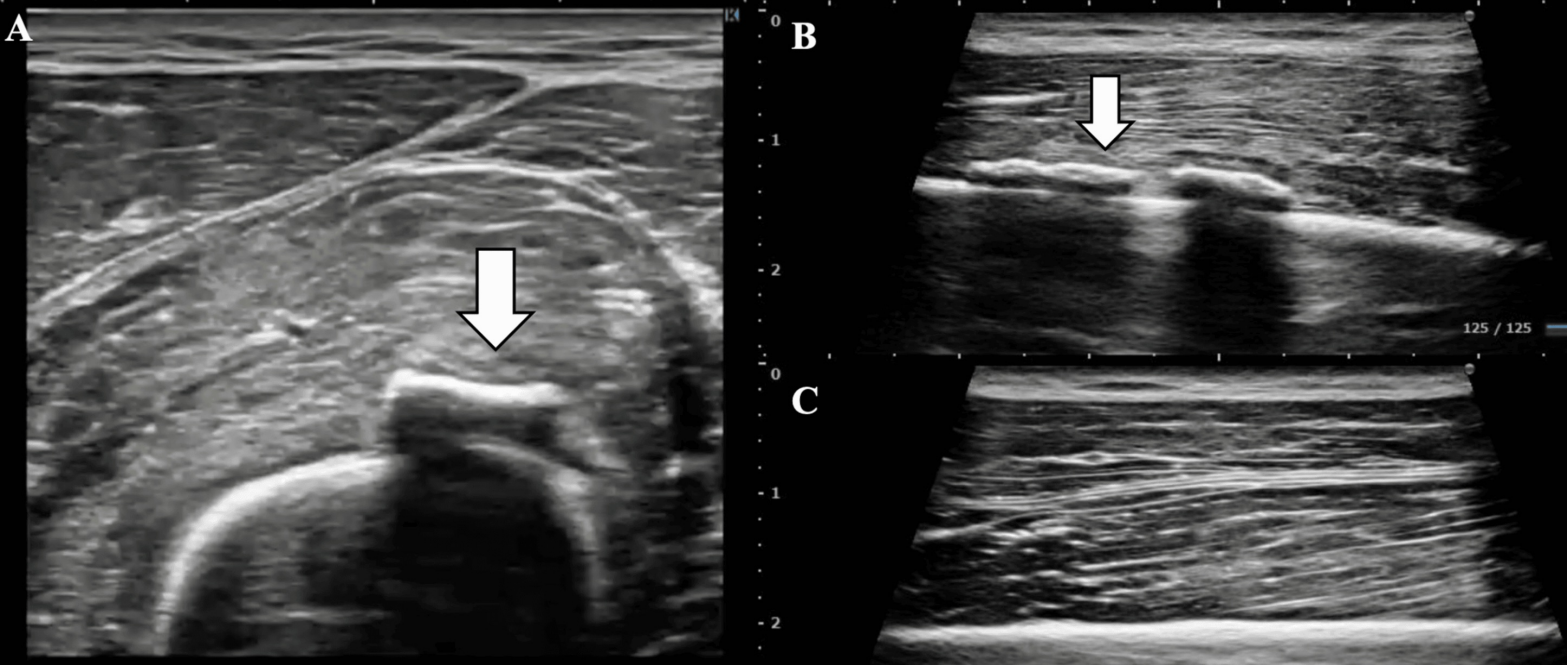
Follow-up ultrasound image of the thigh (A) Short-axis and (B) long-axis ultrasound image of the vastus intermedius on the affected side. (C) Long-axis ultrasound image of the vastus intermedius on the unaffected side Arrow: Heterotopic ossification observed within the vastus intermedius

After four weeks of rest, pain at rest improved, but limitation of knee flexion remained. Reevaluation with ultrasound demonstrated HO at the injured site, and rehabilitation was initiated (Figure 3).

At the initial rehabilitation session, right knee flexion ROM was 120°, with pain localized at the HO site during flexion. Active knee flexion in the prone position was 110°. Dynamic ultrasound evaluation with probe compression demonstrated impaired tissue gliding at the deep surface of the vastus intermedius at the HO site on the affected side compared with the contralateral side (Videos 1-3).

**Video 1:** Dynamic ultrasound of the vastus intermedius (model demonstration) Probe compression was applied to the affected site

**Video 2:** Dynamic ultrasound evaluation of the vastus intermedius on the affected side at the initial rehabilitation session Impaired deep gliding of the vastus intermedius around the heterotopic ossification

**Video 3:** Dynamic ultrasound evaluation of the vastus intermedius on the unaffected side at the initial rehabilitation session Good deep gliding of the vastus intermedius was confirmed compared with the affected side

Ultrasound-guided manual therapy was performed around the HO in the vastus intermedius. The patient was placed in the supine position with the knee extended at 0°. Using a linear probe (10-12 MHz), the HO site was identified. Manual lifting was applied perpendicularly to the femur with moderate pressure for 30 repetitions of five seconds each, followed by transverse gliding for approximately three minutes (Videos 4-7).

**Video 4:** Ultrasound-guided manual therapy (lifting maneuver) demonstrated on a model Performed under monitoring of the affected site with impaired gliding

**Video 5:** Ultrasound video of the affected side during ultrasound-guided manual therapy (lifting maneuver) Manual lifting is applied to separate the deep vastus intermedius with impaired gliding from the heterotopic ossification

**Video 6:** Ultrasound-guided manual therapy (short-axis gliding maneuver) demonstrated on a model Performed under monitoring of the affected site with impaired gliding

**Video 7:** Ultrasound video of the affected side during ultrasound-guided manual therapy (short-axis gliding maneuver) A transverse gliding maneuver was performed while monitoring on ultrasound the deep vastus intermedius at the heterotopic ossification site with impaired gliding to facilitate horizontal sliding

After the first intervention, knee flexion ROM improved to 130°, and pain decreased from 8 to 3 on the Numeric Rating Scale. Dynamic ultrasound evaluation also confirmed improved tissue mobility (Video 8).

**Video 8:** Dynamic ultrasound evaluation of the vastus intermedius on the affected side after the initial rehabilitation session Improved deep gliding of the vastus intermedius after intervention compared with before intervention

A home exercise program was prescribed, in which the patient marked the HO site and performed lifting and gliding maneuvers. Rehabilitation was performed once per week, and after approximately one month (at the end of the fourth session), the patient achieved full pain-free knee flexion and running. In two months, he returned to team training, and rehabilitation was completed (Table 1).

**Table 1:** Clinical course of the patient The table summarizes the progression of knee flexion ROM, NRS, imaging findings, and rehabilitation interventions. Full pain-free flexion and running were achieved within one month after the initiation of rehabilitation, and the patient returned to team training within two months ROM: range of motion; NRS: Numerical Rating Scale; MRI: magnetic resonance imaging; US: ultrasonography; HO: heterotopic ossification

Time from injury	Event/evaluation	Findings (ROM/pain)	Imaging findings	Intervention
Day 0	Direct blow to anterior thigh during soccer match	Severe pain, swelling	-	Initial conservative treatment at local clinic
3 months	Persistent pain and knee flexion limitation	Knee flexion ~120°, pain NRS 8	MRI/US: vastus intermedius contusion	Referral to our hospital
4 months	Before initial rehabilitation intervention	Knee flexion 120° (prone active 110°), pain NRS 8	US: HO and impaired gliding of the vastus intermedius at the HO site	First ultrasound-guided manual therapy session
	After initial rehabilitation intervention	Knee flexion improved to 130°, pain NRS 3	US: improved soft tissue mobility	Home exercise program initiated
5 months	End of fourth rehabilitation session	Full pain-free flexion, able to run	US: maintained tissue gliding	Continued home program
6 months	Return to team training	No symptoms	-	Rehabilitation completed

## Discussion

This case report describes a collegiate soccer player who developed HO in the vastus intermedius following a quadriceps contusion that caused persistent limitation of knee flexion. Ultrasound-guided manual therapy targeting impaired tissue gliding resulted in significant improvements in ROM, pain reduction, and successful return to sports.

Previous studies have reported that conservative treatment for quadriceps HO often requires up to 24 weeks before the patient returns to sports [6,7,10]. In contrast, this athlete returned to training within approximately eight weeks of initiating rehabilitation, suggesting that ultrasound-guided manual therapy may accelerate recovery compared to conventional approaches [10]. However, the possibility that the observed improvement was partly due to the natural healing and maturation process of HO cannot be ruled out, and the therapeutic effect cannot be definitively attributed solely to manual therapy.

The mechanism underlying the favorable outcome in this case was likely not a direct reduction of the HO mass but rather a restoration of soft tissue mobility around the lesion. HO frequently causes adhesions between the ossified lesion and the adjacent muscle or fascia, contributing to pain and motion limitation. Real-time ultrasound enabled the precise identification of the impaired sliding interface and guided targeted manual therapy. This approach reduces mechanical impingement during knee flexion and improves functional mobility. Similar benefits of ultrasound-guided manual therapy have been reported in cases of Achilles tendon adhesion after surgical repair [11], supporting its broader applicability in musculoskeletal rehabilitation. Compared with other treatment strategies, such as pharmacological prophylaxis, shockwave therapy, or surgical excision, ultrasound-guided manual therapy offers a safe, noninvasive, and cost-effective alternative. Importantly, surgical excision is generally delayed for at least six months until the lesion has matured to avoid recurrence [8,9]. Therefore, effective conservative options during the early-to-mid phase of HO are clinically valuable. However, in this case, the therapeutic effect was judged based on changes in ultrasound-observed tissue gliding and clinical symptoms, and without objective quantitative measures, the proposed mechanism remains speculative.

This case highlights the growing role of rehabilitative ultrasound imaging in sports medicine. Ultrasound not only facilitates accurate diagnosis [12,13] but also provides dynamic feedback for guiding manual therapy and patient education [14,15]. Visualization of pathological tissue interfaces may enhance the precision and effectiveness of rehabilitation interventions. However, the use of ultrasound has various limitations, as its effectiveness can be influenced by the environment, the operator’s experience and skills, as well as the location and depth of the lesion, which may sometimes hinder adequate visualization. In addition, the use of ultrasound may be restricted by legal and professional regulations in some countries, where physiotherapists are not permitted to operate diagnostic ultrasound devices.

This study has some limitations. First, HO demonstrates substantial individual variability in size, location, and maturation rate, which limits the generalizability of the findings to a single case. Second, the reproducibility of ultrasound assessment may be limited by factors such as probe fixation and examiner technique. Third, standardized outcome measures such as the International Knee Documentation Committee Subjective Knee Form were not applied, which limited the ability to objectively evaluate knee function.

## Conclusions

This case highlights the potential of ultrasound-guided manual therapy as an effective and noninvasive treatment for motion limitation caused by HO following quadriceps contusions. By providing real-time visualization of tissue adhesions and enabling targeted intervention, this approach facilitated rapid improvement in pain and knee flexion, leading to an earlier return to sports compared with previously reported conservative cases. Ultrasound-guided manual therapy should be considered a valuable conservative option prior to surgical excision, particularly in athletes requiring timely functional recovery.
